# Supplementary material for: Modeling glioblastoma heterogeneity as a dynamic network of cell states
Source: Mol Syst Biol. 2021 Sep 16;17(9):e10105. doi: 10.15252/msb.202010105 (PMC8444284; doi:10.15252/msb.202010105)
Supplement: Supplementary file 5 — Source Data for Figure 3 [file MSB-17-e10105-s001.zip › Figure3A_sourcedata/GSEA_3065/nosigdb_state1.GseaPreranked.1622718409535/SINGH_SOX2_UPREGULATED_TRANSCRIPTS_PMID_28122245.html]

Details for gene set SINGH\_SOX2\_UPREGULATED\_TRANSCRIPTS\_PMID\_28122245[GSEA]

|  || Dataset | state1 |
| Phenotype | NoPhenotypeAvailable |
| Upregulated in class | na\_neg |
| GeneSet | SINGH\_SOX2\_UPREGULATED\_TRANSCRIPTS\_PMID\_28122245 |
| Enrichment Score (ES) | -0.59615993 |
| Normalized Enrichment Score (NES) | -2.4897475 |
| Nominal p-value | 0.0 |
| FDR q-value | 0.0 |
| FWER p-Value | 0.0 |
Table: GSEA Results Summary

  

Fig 1: Enrichment plot: SINGH\_SOX2\_UPREGULATED\_TRANSCRIPTS\_PMID\_28122245      
 Profile of the Running ES Score & Positions of GeneSet Members on the Rank Ordered List

  

| PROBE | GENE SYMBOL | GENE\_TITLE | RANK IN GENE LIST | RANK METRIC SCORE | RUNNING ES | CORE ENRICHMENT || 1 | S100A16 |  |  | 27 | 0.587 | 0.0078 | No |
| 2 | DPYSL3 |  |  | 74 | 0.411 | 0.0104 | No |
| 3 | LSM4 |  |  | 162 | 0.316 | 0.0070 | No |
| 4 | NDUFA6 |  |  | 347 | 0.242 | -0.0081 | No |
| 5 | LSM5 |  |  | 448 | 0.216 | -0.0148 | No |
| 6 | RBM8A |  |  | 464 | 0.213 | -0.0125 | No |
| 7 | TMEM106C |  |  | 478 | 0.211 | -0.0100 | No |
| 8 | MALSU1 |  |  | 493 | 0.207 | -0.0077 | No |
| 9 | LYPD1 |  |  | 508 | 0.205 | -0.0055 | No |
| 10 | TPX2 |  |  | 556 | 0.194 | -0.0069 | No |
| 11 | RPL23A |  |  | 612 | 0.184 | -0.0094 | No |
| 12 | ETV4 |  |  | 665 | 0.176 | -0.0117 | No |
| 13 | MRPL52 |  |  | 805 | 0.156 | -0.0236 | No |
| 14 | MRPL40 |  |  | 838 | 0.153 | -0.0242 | No |
| 15 | AKIRIN2 |  |  | 873 | 0.148 | -0.0251 | No |
| 16 | SMAD1 |  |  | 901 | 0.145 | -0.0253 | No |
| 17 | DCPS |  |  | 1077 | 0.124 | -0.0416 | No |
| 18 | SRP72 |  |  | 1136 | 0.119 | -0.0455 | No |
| 19 | UBALD2 |  |  | 1161 | 0.117 | -0.0459 | No |
| 20 | SPRY2 |  |  | 1171 | 0.115 | -0.0448 | No |
| 21 | HNRNPC |  |  | 1184 | 0.114 | -0.0440 | No |
| 22 | CMSS1 |  |  | 1229 | 0.110 | -0.0467 | No |
| 23 | TAF12 |  |  | 1311 | 0.102 | -0.0534 | No |
| 24 | VAV3 |  |  | 1338 | 0.100 | -0.0543 | No |
| 25 | CENPV |  |  | 1361 | 0.098 | -0.0548 | No |
| 26 | MAD2L1 |  |  | 1394 | 0.095 | -0.0565 | No |
| 27 | THUMPD3 |  |  | 1402 | 0.095 | -0.0555 | No |
| 28 | UBE2T |  |  | 1440 | 0.093 | -0.0577 | No |
| 29 | NUCKS1 |  |  | 1540 | 0.087 | -0.0666 | No |
| 30 | AIMP2 |  |  | 1560 | 0.085 | -0.0671 | No |
| 31 | SKA2 |  |  | 1599 | 0.082 | -0.0696 | No |
| 32 | HIST1H1E |  |  | 1608 | 0.082 | -0.0690 | No |
| 33 | MRPS14 |  |  | 1672 | 0.077 | -0.0742 | No |
| 34 | ANP32E |  |  | 1706 | 0.075 | -0.0764 | No |
| 35 | MRPS21 |  |  | 1739 | 0.073 | -0.0784 | No |
| 36 | CENPA |  |  | 1788 | 0.070 | -0.0822 | No |
| 37 | AGK |  |  | 1840 | 0.068 | -0.0864 | No |
| 38 | RAD51C |  |  | 1874 | 0.066 | -0.0887 | No |
| 39 | RPF1 |  |  | 1896 | 0.065 | -0.0897 | No |
| 40 | USP1 |  |  | 1921 | 0.064 | -0.0911 | No |
| 41 | ACD |  |  | 1998 | 0.060 | -0.0980 | No |
| 42 | DENND2A |  |  | 2043 | 0.057 | -0.1016 | No |
| 43 | XPO6 |  |  | 2053 | 0.057 | -0.1016 | No |
| 44 | SRI |  |  | 2084 | 0.056 | -0.1037 | No |
| 45 | HNRNPUL1 |  |  | 2106 | 0.055 | -0.1049 | No |
| 46 | PEF1 |  |  | 2233 | 0.050 | -0.1174 | No |
| 47 | FAM192A |  |  | 2278 | 0.048 | -0.1212 | No |
| 48 | DLAT |  |  | 2282 | 0.048 | -0.1206 | No |
| 49 | NDE1 |  |  | 2284 | 0.048 | -0.1198 | No |
| 50 | PRR11 |  |  | 2329 | 0.046 | -0.1237 | No |
| 51 | FAM84B |  |  | 2432 | 0.042 | -0.1337 | No |
| 52 | PAICS |  |  | 2456 | 0.041 | -0.1354 | No |
| 53 | ARMC6 |  |  | 2556 | 0.038 | -0.1452 | No |
| 54 | FBXL7 |  |  | 2568 | 0.038 | -0.1456 | No |
| 55 | APOO |  |  | 2641 | 0.035 | -0.1526 | No |
| 56 | RAD23B |  |  | 2654 | 0.034 | -0.1533 | No |
| 57 | STX5 |  |  | 2693 | 0.033 | -0.1567 | No |
| 58 | HIST1H1C |  |  | 2712 | 0.033 | -0.1580 | No |
| 59 | PPIL1 |  |  | 2749 | 0.031 | -0.1612 | No |
| 60 | ZC3HC1 |  |  | 2809 | 0.030 | -0.1669 | No |
| 61 | OSGIN2 |  |  | 2849 | 0.029 | -0.1705 | No |
| 62 | FBXO5 |  |  | 2870 | 0.028 | -0.1721 | No |
| 63 | ZBTB2 |  |  | 2880 | 0.028 | -0.1726 | No |
| 64 | BUB1 |  |  | 2888 | 0.028 | -0.1728 | No |
| 65 | ELP5 |  |  | 2974 | 0.026 | -0.1813 | No |
| 66 | EPS15L1 |  |  | 2984 | 0.025 | -0.1818 | No |
| 67 | RGMA |  |  | 3071 | 0.023 | -0.1905 | No |
| 68 | TLK1 |  |  | 3089 | 0.023 | -0.1919 | No |
| 69 | TMPO |  |  | 3093 | 0.023 | -0.1918 | No |
| 70 | PPAT |  |  | 3095 | 0.023 | -0.1915 | No |
| 71 | STX8 |  |  | 3115 | 0.022 | -0.1931 | No |
| 72 | DARS |  |  | 3142 | 0.022 | -0.1955 | No |
| 73 | CHIC2 |  |  | 3170 | 0.021 | -0.1979 | No |
| 74 | RPA1 |  |  | 3239 | 0.020 | -0.2048 | No |
| 75 | CTNNBL1 |  |  | 3282 | 0.019 | -0.2089 | No |
| 76 | LYRM2 |  |  | 3289 | 0.018 | -0.2092 | No |
| 77 | PPP2R1A |  |  | 3309 | 0.018 | -0.2109 | No |
| 78 | TMEM177 |  |  | 3350 | 0.017 | -0.2148 | No |
| 79 | ZNF770 |  |  | 3353 | 0.017 | -0.2147 | No |
| 80 | CHTOP |  |  | 3387 | 0.016 | -0.2179 | No |
| 81 | CDCA7L |  |  | 3390 | 0.016 | -0.2178 | No |
| 82 | CDK1 |  |  | 3396 | 0.016 | -0.2180 | No |
| 83 | ZFR |  |  | 3455 | 0.015 | -0.2239 | No |
| 84 | ZNF205 |  |  | 3462 | 0.015 | -0.2243 | No |
| 85 | AJUBA |  |  | 3503 | 0.014 | -0.2283 | No |
| 86 | NHSL1 |  |  | 3531 | 0.013 | -0.2309 | No |
| 87 | TTC28 |  |  | 3597 | 0.012 | -0.2375 | No |
| 88 | PI4KB |  |  | 3602 | 0.012 | -0.2377 | No |
| 89 | HIST1H1D |  |  | 3604 | 0.012 | -0.2376 | No |
| 90 | DDX42 |  |  | 3634 | 0.011 | -0.2405 | No |
| 91 | REPS1 |  |  | 3641 | 0.011 | -0.2409 | No |
| 92 | SP3 |  |  | 3699 | 0.010 | -0.2468 | No |
| 93 | MED19 |  |  | 3720 | 0.009 | -0.2487 | No |
| 94 | WDR82 |  |  | 3772 | 0.009 | -0.2540 | No |
| 95 | PTPRS |  |  | 3773 | 0.009 | -0.2538 | No |
| 96 | TCHP |  |  | 3793 | 0.008 | -0.2557 | No |
| 97 | USP6NL |  |  | 3806 | 0.008 | -0.2568 | No |
| 98 | NXF1 |  |  | 3809 | 0.008 | -0.2569 | No |
| 99 | CUEDC1 |  |  | 3852 | 0.007 | -0.2612 | No |
| 100 | PRPF19 |  |  | 3862 | 0.007 | -0.2620 | No |
| 101 | ETV5 |  |  | 3894 | 0.006 | -0.2652 | No |
| 102 | WRAP53 |  |  | 3898 | 0.006 | -0.2654 | No |
| 103 | CCDC112 |  |  | 3982 | 0.005 | -0.2741 | No |
| 104 | FUZ |  |  | 4022 | 0.004 | -0.2781 | No |
| 105 | ARNTL |  |  | 4033 | 0.004 | -0.2791 | No |
| 106 | RAB8B |  |  | 4042 | 0.003 | -0.2799 | No |
| 107 | COA1 |  |  | 4046 | 0.003 | -0.2802 | No |
| 108 | NEK1 |  |  | 4071 | 0.003 | -0.2827 | No |
| 109 | LIX1L |  |  | 4097 | 0.003 | -0.2852 | No |
| 110 | EXOSC2 |  |  | 4141 | 0.002 | -0.2898 | No |
| 111 | HIST1H2AC |  |  | 4158 | 0.001 | -0.2914 | No |
| 112 | ZFP64 |  |  | 4194 | 0.001 | -0.2951 | No |
| 113 | DPF3 |  |  | 4225 | 0.000 | -0.2983 | No |
| 114 | NCAPG |  |  | 4261 | -0.000 | -0.3020 | No |
| 115 | FAM118B |  |  | 4286 | -0.001 | -0.3045 | No |
| 116 | PSAT1 |  |  | 4318 | -0.001 | -0.3078 | No |
| 117 | PRMT5 |  |  | 4376 | -0.002 | -0.3138 | No |
| 118 | HARS2 |  |  | 4392 | -0.003 | -0.3153 | No |
| 119 | DNAJA3 |  |  | 4475 | -0.004 | -0.3239 | No |
| 120 | BUD13 |  |  | 4500 | -0.004 | -0.3264 | No |
| 121 | RIOK2 |  |  | 4501 | -0.004 | -0.3263 | No |
| 122 | RNF214 |  |  | 4556 | -0.006 | -0.3319 | No |
| 123 | BMP7 |  |  | 4562 | -0.006 | -0.3323 | No |
| 124 | GCFC2 |  |  | 4564 | -0.006 | -0.3323 | No |
| 125 | CDKN1B |  |  | 4655 | -0.007 | -0.3417 | No |
| 126 | ZSCAN16 |  |  | 4659 | -0.007 | -0.3419 | No |
| 127 | VPS72 |  |  | 4685 | -0.007 | -0.3444 | No |
| 128 | CCDC97 |  |  | 4695 | -0.007 | -0.3452 | No |
| 129 | SH3BP4 |  |  | 4709 | -0.008 | -0.3465 | No |
| 130 | AURKB |  |  | 4764 | -0.008 | -0.3520 | No |
| 131 | RPUSD4 |  |  | 4797 | -0.009 | -0.3553 | No |
| 132 | NR3C1 |  |  | 4804 | -0.009 | -0.3557 | No |
| 133 | ZNF326 |  |  | 4817 | -0.009 | -0.3568 | No |
| 134 | SOX21 |  |  | 4829 | -0.010 | -0.3578 | No |
| 135 | ELMO2 |  |  | 4842 | -0.010 | -0.3589 | No |
| 136 | ZMYND11 |  |  | 4854 | -0.010 | -0.3599 | No |
| 137 | ADK |  |  | 4909 | -0.011 | -0.3654 | No |
| 138 | EDC3 |  |  | 4915 | -0.011 | -0.3657 | No |
| 139 | RAD21 |  |  | 4916 | -0.011 | -0.3655 | No |
| 140 | NDP |  |  | 4946 | -0.012 | -0.3684 | No |
| 141 | OSBPL6 |  |  | 4965 | -0.012 | -0.3701 | No |
| 142 | GTF2IRD1 |  |  | 5049 | -0.013 | -0.3786 | No |
| 143 | BANP |  |  | 5052 | -0.013 | -0.3786 | No |
| 144 | SEC22C |  |  | 5077 | -0.014 | -0.3809 | No |
| 145 | HIST1H1B |  |  | 5125 | -0.014 | -0.3856 | No |
| 146 | PDIK1L |  |  | 5129 | -0.014 | -0.3856 | No |
| 147 | DVL2 |  |  | 5158 | -0.015 | -0.3883 | No |
| 148 | DOK5 |  |  | 5166 | -0.015 | -0.3888 | No |
| 149 | KLC4 |  |  | 5194 | -0.015 | -0.3914 | No |
| 150 | TRAPPC9 |  |  | 5204 | -0.015 | -0.3920 | No |
| 151 | PFAS |  |  | 5208 | -0.016 | -0.3921 | No |
| 152 | MSL2 |  |  | 5213 | -0.016 | -0.3922 | No |
| 153 | PBX3 |  |  | 5220 | -0.016 | -0.3926 | No |
| 154 | KIF14 |  |  | 5262 | -0.016 | -0.3966 | No |
| 155 | ZNF614 |  |  | 5292 | -0.017 | -0.3994 | No |
| 156 | CCDC34 |  |  | 5298 | -0.017 | -0.3996 | No |
| 157 | WIPF2 |  |  | 5308 | -0.017 | -0.4002 | No |
| 158 | MBIP |  |  | 5359 | -0.018 | -0.4052 | No |
| 159 | USP3 |  |  | 5375 | -0.018 | -0.4065 | No |
| 160 | CPSF7 |  |  | 5434 | -0.019 | -0.4123 | No |
| 161 | KPNA2 |  |  | 5461 | -0.020 | -0.4147 | No |
| 162 | ZBED5 |  |  | 5507 | -0.020 | -0.4190 | No |
| 163 | OPA1 |  |  | 5522 | -0.020 | -0.4202 | No |
| 164 | TP53 |  |  | 5557 | -0.021 | -0.4234 | No |
| 165 | MTHFD1 |  |  | 5596 | -0.022 | -0.4270 | No |
| 166 | NVL |  |  | 5614 | -0.022 | -0.4284 | No |
| 167 | ZNF687 |  |  | 5617 | -0.022 | -0.4282 | No |
| 168 | MED25 |  |  | 5659 | -0.023 | -0.4321 | No |
| 169 | SPATA5L1 |  |  | 5700 | -0.024 | -0.4359 | No |
| 170 | KIF2A |  |  | 5712 | -0.024 | -0.4367 | No |
| 171 | C2orf76 |  |  | 5715 | -0.024 | -0.4364 | No |
| 172 | ITGB3BP |  |  | 5717 | -0.024 | -0.4361 | No |
| 173 | GAPVD1 |  |  | 5766 | -0.025 | -0.4407 | No |
| 174 | VPS37B |  |  | 5829 | -0.026 | -0.4468 | No |
| 175 | ENKD1 |  |  | 5862 | -0.026 | -0.4497 | No |
| 176 | PITPNC1 |  |  | 5863 | -0.026 | -0.4492 | No |
| 177 | PTOV1 |  |  | 5870 | -0.027 | -0.4494 | No |
| 178 | DAB2IP |  |  | 5878 | -0.027 | -0.4496 | No |
| 179 | CDYL |  |  | 5881 | -0.027 | -0.4494 | No |
| 180 | RMI1 |  |  | 5899 | -0.027 | -0.4507 | No |
| 181 | MCCC2 |  |  | 5900 | -0.027 | -0.4502 | No |
| 182 | SASS6 |  |  | 5909 | -0.028 | -0.4505 | No |
| 183 | PCCB |  |  | 5924 | -0.028 | -0.4515 | No |
| 184 | C16orf87 |  |  | 5943 | -0.028 | -0.4529 | No |
| 185 | TSEN15 |  |  | 5974 | -0.029 | -0.4555 | No |
| 186 | WDR47 |  |  | 5977 | -0.029 | -0.4552 | No |
| 187 | CWC25 |  |  | 5981 | -0.029 | -0.4550 | No |
| 188 | UBXN2B |  |  | 5992 | -0.029 | -0.4555 | No |
| 189 | DHX8 |  |  | 6008 | -0.029 | -0.4566 | No |
| 190 | ZBED3 |  |  | 6039 | -0.030 | -0.4592 | No |
| 191 | KIAA1147 |  |  | 6106 | -0.031 | -0.4656 | No |
| 192 | FBXW11 |  |  | 6201 | -0.033 | -0.4750 | No |
| 193 | AP1G1 |  |  | 6204 | -0.033 | -0.4746 | No |
| 194 | FOXG1 |  |  | 6245 | -0.034 | -0.4782 | No |
| 195 | CDKAL1 |  |  | 6308 | -0.035 | -0.4841 | No |
| 196 | NBR1 |  |  | 6358 | -0.036 | -0.4887 | No |
| 197 | PHF12 |  |  | 6432 | -0.037 | -0.4957 | No |
| 198 | KAT6B |  |  | 6433 | -0.037 | -0.4951 | No |
| 199 | ZNF521 |  |  | 6445 | -0.037 | -0.4955 | No |
| 200 | SP4 |  |  | 6473 | -0.038 | -0.4977 | No |
| 201 | TSN |  |  | 6557 | -0.040 | -0.5058 | No |
| 202 | SCHIP1 |  |  | 6575 | -0.040 | -0.5068 | No |
| 203 | DCAF16 |  |  | 6590 | -0.040 | -0.5076 | No |
| 204 | PPM1D |  |  | 6609 | -0.041 | -0.5087 | No |
| 205 | AP3M1 |  |  | 6659 | -0.042 | -0.5132 | No |
| 206 | ERCC4 |  |  | 6735 | -0.044 | -0.5203 | No |
| 207 | ZC3H4 |  |  | 6742 | -0.044 | -0.5201 | No |
| 208 | RBM23 |  |  | 6744 | -0.044 | -0.5195 | No |
| 209 | DCAF12 |  |  | 6767 | -0.044 | -0.5210 | No |
| 210 | ERCC2 |  |  | 6845 | -0.046 | -0.5283 | No |
| 211 | ZNF507 |  |  | 6875 | -0.046 | -0.5305 | No |
| 212 | CNOT2 |  |  | 6899 | -0.047 | -0.5321 | No |
| 213 | ARFGAP2 |  |  | 6918 | -0.048 | -0.5331 | No |
| 214 | DDAH1 |  |  | 6943 | -0.049 | -0.5348 | No |
| 215 | SPATA5 |  |  | 6971 | -0.049 | -0.5367 | No |
| 216 | ZNF350 |  |  | 6980 | -0.049 | -0.5367 | No |
| 217 | RNF38 |  |  | 6996 | -0.050 | -0.5374 | No |
| 218 | E2F6 |  |  | 7014 | -0.050 | -0.5382 | No |
| 219 | ELP3 |  |  | 7071 | -0.052 | -0.5432 | No |
| 220 | IRAK1BP1 |  |  | 7088 | -0.053 | -0.5440 | No |
| 221 | DCAKD |  |  | 7092 | -0.053 | -0.5433 | No |
| 222 | COMMD2 |  |  | 7125 | -0.054 | -0.5457 | No |
| 223 | FAN1 |  |  | 7157 | -0.054 | -0.5480 | No |
| 224 | PTK2 |  |  | 7170 | -0.055 | -0.5483 | No |
| 225 | CLDN12 |  |  | 7198 | -0.055 | -0.5501 | No |
| 226 | FAM110B |  |  | 7201 | -0.055 | -0.5493 | No |
| 227 | HERPUD2 |  |  | 7236 | -0.056 | -0.5519 | No |
| 228 | EZH2 |  |  | 7260 | -0.057 | -0.5533 | No |
| 229 | RFX2 |  |  | 7265 | -0.057 | -0.5527 | No |
| 230 | POGK |  |  | 7267 | -0.057 | -0.5518 | No |
| 231 | ZNF649 |  |  | 7352 | -0.059 | -0.5596 | No |
| 232 | C11orf49 |  |  | 7379 | -0.060 | -0.5612 | No |
| 233 | TLCD1 |  |  | 7387 | -0.060 | -0.5609 | No |
| 234 | DUSP16 |  |  | 7416 | -0.061 | -0.5627 | No |
| 235 | SMARCAD1 |  |  | 7418 | -0.061 | -0.5617 | No |
| 236 | AASDH |  |  | 7420 | -0.061 | -0.5607 | No |
| 237 | JAG1 |  |  | 7426 | -0.061 | -0.5602 | No |
| 238 | GLI3 |  |  | 7475 | -0.062 | -0.5641 | No |
| 239 | TRAF4 |  |  | 7503 | -0.064 | -0.5658 | No |
| 240 | CCDC80 |  |  | 7525 | -0.064 | -0.5669 | No |
| 241 | USP24 |  |  | 7529 | -0.064 | -0.5660 | No |
| 242 | ZNF626 |  |  | 7567 | -0.065 | -0.5687 | No |
| 243 | PLCD3 |  |  | 7614 | -0.067 | -0.5724 | No |
| 244 | C6orf89 |  |  | 7621 | -0.067 | -0.5718 | No |
| 245 | ZHX3 |  |  | 7622 | -0.067 | -0.5706 | No |
| 246 | CEP78 |  |  | 7627 | -0.067 | -0.5698 | No |
| 247 | KAT6A |  |  | 7636 | -0.067 | -0.5694 | No |
| 248 | PARP11 |  |  | 7637 | -0.067 | -0.5682 | No |
| 249 | ARHGAP42 |  |  | 7652 | -0.068 | -0.5684 | No |
| 250 | ZNF814 |  |  | 7695 | -0.070 | -0.5716 | No |
| 251 | TMX2 |  |  | 7722 | -0.071 | -0.5731 | No |
| 252 | CDC45 |  |  | 7751 | -0.072 | -0.5747 | No |
| 253 | LFNG |  |  | 7784 | -0.073 | -0.5768 | No |
| 254 | TNRC6C |  |  | 7785 | -0.073 | -0.5755 | No |
| 255 | NAALADL2 |  |  | 7842 | -0.075 | -0.5800 | No |
| 256 | RIMKLB |  |  | 7846 | -0.075 | -0.5790 | No |
| 257 | ZNF608 |  |  | 7855 | -0.075 | -0.5785 | No |
| 258 | XPO1 |  |  | 7863 | -0.076 | -0.5778 | No |
| 259 | BBS2 |  |  | 7875 | -0.076 | -0.5776 | No |
| 260 | TNIK |  |  | 7901 | -0.077 | -0.5788 | No |
| 261 | ADAMTS6 |  |  | 7912 | -0.078 | -0.5785 | No |
| 262 | NXPH1 |  |  | 7920 | -0.078 | -0.5778 | No |
| 263 | SFXN1 |  |  | 7952 | -0.079 | -0.5796 | No |
| 264 | TUBA1A |  |  | 7955 | -0.080 | -0.5784 | No |
| 265 | NUP205 |  |  | 7962 | -0.080 | -0.5776 | No |
| 266 | RNFT2 |  |  | 8084 | -0.084 | -0.5889 | No |
| 267 | ZNF432 |  |  | 8085 | -0.084 | -0.5873 | No |
| 268 | CTNND1 |  |  | 8094 | -0.085 | -0.5866 | No |
| 269 | BCL7A |  |  | 8103 | -0.085 | -0.5859 | No |
| 270 | ARHGAP32 |  |  | 8122 | -0.086 | -0.5863 | No |
| 271 | CDC14B |  |  | 8123 | -0.086 | -0.5847 | No |
| 272 | DOCK7 |  |  | 8139 | -0.087 | -0.5847 | No |
| 273 | GSTA4 |  |  | 8147 | -0.087 | -0.5839 | No |
| 274 | TLE3 |  |  | 8155 | -0.087 | -0.5830 | No |
| 275 | ZBTB10 |  |  | 8173 | -0.089 | -0.5832 | No |
| 276 | EPC1 |  |  | 8193 | -0.089 | -0.5836 | No |
| 277 | FTO |  |  | 8194 | -0.089 | -0.5820 | No |
| 278 | NEDD1 |  |  | 8208 | -0.090 | -0.5817 | No |
| 279 | ZNF268 |  |  | 8214 | -0.090 | -0.5806 | No |
| 280 | PUM1 |  |  | 8221 | -0.090 | -0.5796 | No |
| 281 | IL17RD |  |  | 8242 | -0.091 | -0.5800 | No |
| 282 | CDH4 |  |  | 8271 | -0.093 | -0.5813 | No |
| 283 | C1orf56 |  |  | 8272 | -0.093 | -0.5796 | No |
| 284 | NCKAP5 |  |  | 8280 | -0.094 | -0.5787 | No |
| 285 | PNPT1 |  |  | 8313 | -0.095 | -0.5803 | No |
| 286 | MPDZ |  |  | 8337 | -0.096 | -0.5810 | No |
| 287 | PMS2 |  |  | 8350 | -0.097 | -0.5805 | No |
| 288 | TNFRSF19 |  |  | 8355 | -0.097 | -0.5792 | No |
| 289 | GPT2 |  |  | 8405 | -0.099 | -0.5825 | No |
| 290 | ZNF680 |  |  | 8408 | -0.100 | -0.5809 | No |
| 291 | AQR |  |  | 8440 | -0.101 | -0.5824 | No |
| 292 | LCORL |  |  | 8458 | -0.102 | -0.5823 | No |
| 293 | CDK5RAP2 |  |  | 8496 | -0.105 | -0.5843 | No |
| 294 | CASK |  |  | 8532 | -0.107 | -0.5861 | No |
| 295 | APBA2 |  |  | 8541 | -0.108 | -0.5850 | No |
| 296 | AUTS2 |  |  | 8583 | -0.111 | -0.5873 | No |
| 297 | MTF2 |  |  | 8595 | -0.111 | -0.5864 | No |
| 298 | TCF12 |  |  | 8614 | -0.112 | -0.5863 | No |
| 299 | PKN2 |  |  | 8647 | -0.114 | -0.5876 | No |
| 300 | RFX3 |  |  | 8690 | -0.117 | -0.5899 | No |
| 301 | MAP3K1 |  |  | 8750 | -0.121 | -0.5940 | Yes |
| 302 | DNAJC18 |  |  | 8753 | -0.122 | -0.5919 | Yes |
| 303 | SMARCA4 |  |  | 8760 | -0.122 | -0.5904 | Yes |
| 304 | PTCH1 |  |  | 8777 | -0.123 | -0.5898 | Yes |
| 305 | ARHGAP35 |  |  | 8793 | -0.124 | -0.5891 | Yes |
| 306 | HIST1H4C |  |  | 8820 | -0.126 | -0.5896 | Yes |
| 307 | POU2F1 |  |  | 8841 | -0.128 | -0.5894 | Yes |
| 308 | SUCO |  |  | 8851 | -0.128 | -0.5880 | Yes |
| 309 | ID4 |  |  | 8856 | -0.129 | -0.5861 | Yes |
| 310 | ARHGAP31 |  |  | 8865 | -0.130 | -0.5846 | Yes |
| 311 | DHX36 |  |  | 8895 | -0.134 | -0.5852 | Yes |
| 312 | DHX40 |  |  | 8906 | -0.134 | -0.5838 | Yes |
| 313 | CNOT4 |  |  | 8916 | -0.136 | -0.5823 | Yes |
| 314 | FANCI |  |  | 8917 | -0.136 | -0.5798 | Yes |
| 315 | TMEM209 |  |  | 8952 | -0.139 | -0.5809 | Yes |
| 316 | SHC3 |  |  | 8972 | -0.141 | -0.5804 | Yes |
| 317 | UBR5 |  |  | 8995 | -0.142 | -0.5801 | Yes |
| 318 | VANGL2 |  |  | 9001 | -0.142 | -0.5780 | Yes |
| 319 | KIAA1958 |  |  | 9015 | -0.144 | -0.5768 | Yes |
| 320 | PBX1 |  |  | 9047 | -0.147 | -0.5774 | Yes |
| 321 | PTAR1 |  |  | 9056 | -0.148 | -0.5756 | Yes |
| 322 | TMEM33 |  |  | 9066 | -0.149 | -0.5738 | Yes |
| 323 | SALL1 |  |  | 9084 | -0.151 | -0.5729 | Yes |
| 324 | STRADA |  |  | 9089 | -0.151 | -0.5705 | Yes |
| 325 | SOX5 |  |  | 9104 | -0.152 | -0.5693 | Yes |
| 326 | KIF21A |  |  | 9110 | -0.153 | -0.5670 | Yes |
| 327 | KLHDC8A |  |  | 9117 | -0.153 | -0.5648 | Yes |
| 328 | IGSF11 |  |  | 9127 | -0.155 | -0.5630 | Yes |
| 329 | NLGN1 |  |  | 9131 | -0.155 | -0.5605 | Yes |
| 330 | GAB1 |  |  | 9132 | -0.156 | -0.5576 | Yes |
| 331 | PLCE1 |  |  | 9140 | -0.157 | -0.5555 | Yes |
| 332 | POU3F2 |  |  | 9173 | -0.162 | -0.5560 | Yes |
| 333 | NPAS3 |  |  | 9181 | -0.163 | -0.5538 | Yes |
| 334 | CDH2 |  |  | 9187 | -0.163 | -0.5513 | Yes |
| 335 | OPHN1 |  |  | 9189 | -0.164 | -0.5484 | Yes |
| 336 | RNF157 |  |  | 9191 | -0.164 | -0.5456 | Yes |
| 337 | GON4L |  |  | 9236 | -0.171 | -0.5471 | Yes |
| 338 | CELSR2 |  |  | 9238 | -0.171 | -0.5441 | Yes |
| 339 | LRIG1 |  |  | 9256 | -0.173 | -0.5428 | Yes |
| 340 | FAM133B |  |  | 9263 | -0.174 | -0.5402 | Yes |
| 341 | FIGN |  |  | 9275 | -0.176 | -0.5382 | Yes |
| 342 | ARHGEF26 |  |  | 9286 | -0.178 | -0.5360 | Yes |
| 343 | EXOC4 |  |  | 9315 | -0.183 | -0.5356 | Yes |
| 344 | PAG1 |  |  | 9322 | -0.185 | -0.5329 | Yes |
| 345 | TBL1XR1 |  |  | 9339 | -0.187 | -0.5312 | Yes |
| 346 | SPEN |  |  | 9342 | -0.188 | -0.5280 | Yes |
| 347 | TAOK3 |  |  | 9351 | -0.190 | -0.5254 | Yes |
| 348 | ATF7IP |  |  | 9369 | -0.194 | -0.5237 | Yes |
| 349 | RFTN2 |  |  | 9402 | -0.200 | -0.5234 | Yes |
| 350 | MAGED1 |  |  | 9440 | -0.209 | -0.5235 | Yes |
| 351 | NUPL2 |  |  | 9448 | -0.213 | -0.5204 | Yes |
| 352 | PHLPP1 |  |  | 9463 | -0.217 | -0.5179 | Yes |
| 353 | MKLN1 |  |  | 9467 | -0.218 | -0.5143 | Yes |
| 354 | SPECC1 |  |  | 9470 | -0.219 | -0.5105 | Yes |
| 355 | PIK3R1 |  |  | 9474 | -0.220 | -0.5068 | Yes |
| 356 | ZNF462 |  |  | 9480 | -0.222 | -0.5033 | Yes |
| 357 | GOLM1 |  |  | 9481 | -0.222 | -0.4993 | Yes |
| 358 | SPAG9 |  |  | 9487 | -0.223 | -0.4958 | Yes |
| 359 | ZEB1 |  |  | 9492 | -0.224 | -0.4921 | Yes |
| 360 | MSI2 |  |  | 9498 | -0.225 | -0.4885 | Yes |
| 361 | FOXO3 |  |  | 9503 | -0.226 | -0.4848 | Yes |
| 362 | TRIM9 |  |  | 9512 | -0.229 | -0.4815 | Yes |
| 363 | SRGAP1 |  |  | 9518 | -0.230 | -0.4779 | Yes |
| 364 | IPO9 |  |  | 9531 | -0.233 | -0.4749 | Yes |
| 365 | EIF4G3 |  |  | 9556 | -0.242 | -0.4730 | Yes |
| 366 | POGZ |  |  | 9557 | -0.244 | -0.4686 | Yes |
| 367 | TRIM24 |  |  | 9561 | -0.245 | -0.4644 | Yes |
| 368 | LDLRAD3 |  |  | 9567 | -0.247 | -0.4605 | Yes |
| 369 | NFIB |  |  | 9572 | -0.249 | -0.4564 | Yes |
| 370 | FYN |  |  | 9575 | -0.250 | -0.4520 | Yes |
| 371 | APC |  |  | 9577 | -0.251 | -0.4476 | Yes |
| 372 | GLCCI1 |  |  | 9579 | -0.253 | -0.4431 | Yes |
| 373 | JAKMIP2 |  |  | 9583 | -0.254 | -0.4388 | Yes |
| 374 | TJP1 |  |  | 9592 | -0.257 | -0.4350 | Yes |
| 375 | CTNND2 |  |  | 9595 | -0.257 | -0.4305 | Yes |
| 376 | BRCA1 |  |  | 9600 | -0.258 | -0.4262 | Yes |
| 377 | DPP6 |  |  | 9606 | -0.262 | -0.4220 | Yes |
| 378 | CREB5 |  |  | 9616 | -0.265 | -0.4181 | Yes |
| 379 | SUGP2 |  |  | 9618 | -0.265 | -0.4134 | Yes |
| 380 | PTPRA |  |  | 9624 | -0.268 | -0.4091 | Yes |
| 381 | NES |  |  | 9626 | -0.270 | -0.4043 | Yes |
| 382 | SEZ6L |  |  | 9628 | -0.272 | -0.3994 | Yes |
| 383 | NKAIN3 |  |  | 9635 | -0.274 | -0.3951 | Yes |
| 384 | HMGCR |  |  | 9640 | -0.277 | -0.3905 | Yes |
| 385 | RGS12 |  |  | 9644 | -0.277 | -0.3858 | Yes |
| 386 | APBB2 |  |  | 9645 | -0.277 | -0.3807 | Yes |
| 387 | STIM2 |  |  | 9648 | -0.282 | -0.3758 | Yes |
| 388 | KIF1B |  |  | 9649 | -0.282 | -0.3707 | Yes |
| 389 | RFX4 |  |  | 9655 | -0.285 | -0.3660 | Yes |
| 390 | SPATA6 |  |  | 9657 | -0.286 | -0.3609 | Yes |
| 391 | SETD5 |  |  | 9663 | -0.291 | -0.3562 | Yes |
| 392 | CCDC47 |  |  | 9664 | -0.291 | -0.3509 | Yes |
| 393 | TANC2 |  |  | 9668 | -0.293 | -0.3459 | Yes |
| 394 | SOX2 |  |  | 9680 | -0.298 | -0.3416 | Yes |
| 395 | SEMA6A |  |  | 9690 | -0.304 | -0.3370 | Yes |
| 396 | SOX4 |  |  | 9704 | -0.318 | -0.3326 | Yes |
| 397 | DCLK2 |  |  | 9717 | -0.328 | -0.3279 | Yes |
| 398 | HMGCS1 |  |  | 9718 | -0.329 | -0.3220 | Yes |
| 399 | MLC1 |  |  | 9728 | -0.338 | -0.3168 | Yes |
| 400 | SOX11 |  |  | 9730 | -0.339 | -0.3107 | Yes |
| 401 | SF1 |  |  | 9735 | -0.344 | -0.3048 | Yes |
| 402 | ZBTB20 |  |  | 9743 | -0.352 | -0.2992 | Yes |
| 403 | SOX9 |  |  | 9754 | -0.358 | -0.2937 | Yes |
| 404 | MARCKS |  |  | 9763 | -0.370 | -0.2878 | Yes |
| 405 | ETV1 |  |  | 9764 | -0.371 | -0.2811 | Yes |
| 406 | TCF4 |  |  | 9768 | -0.377 | -0.2745 | Yes |
| 407 | OLIG2 |  |  | 9776 | -0.386 | -0.2683 | Yes |
| 408 | SYT11 |  |  | 9785 | -0.399 | -0.2619 | Yes |
| 409 | ITGB8 |  |  | 9794 | -0.418 | -0.2551 | Yes |
| 410 | QKI |  |  | 9803 | -0.430 | -0.2481 | Yes |
| 411 | WSCD1 |  |  | 9808 | -0.448 | -0.2404 | Yes |
| 412 | C1orf61 |  |  | 9813 | -0.479 | -0.2321 | Yes |
| 413 | LSAMP |  |  | 9814 | -0.481 | -0.2234 | Yes |
| 414 | GPM6A |  |  | 9820 | -0.499 | -0.2148 | Yes |
| 415 | EDNRB |  |  | 9824 | -0.509 | -0.2058 | Yes |
| 416 | LINC00461 |  |  | 9830 | -0.527 | -0.1968 | Yes |
| 417 | SCD5 |  |  | 9838 | -0.576 | -0.1871 | Yes |
| 418 | CCND2 |  |  | 9842 | -0.611 | -0.1763 | Yes |
| 419 | OLIG1 |  |  | 9845 | -0.637 | -0.1649 | Yes |
| 420 | FAM181B |  |  | 9854 | -0.697 | -0.1531 | Yes |
| 421 | MAP2 |  |  | 9857 | -0.735 | -0.1399 | Yes |
| 422 | NOVA1 |  |  | 9858 | -0.741 | -0.1264 | Yes |
| 423 | NCAM1 |  |  | 9862 | -0.751 | -0.1131 | Yes |
| 424 | SOX6 |  |  | 9863 | -0.798 | -0.0986 | Yes |
| 425 | TUBB2B |  |  | 9864 | -0.809 | -0.0838 | Yes |
| 426 | PTPRZ1 |  |  | 9869 | -0.918 | -0.0676 | Yes |
| 427 | PTN |  |  | 9874 | -1.107 | -0.0479 | Yes |
| 428 | BCAN |  |  | 9878 | -1.175 | -0.0268 | Yes |
| 429 | SCRG1 |  |  | 9879 | -1.473 | 0.0000 | Yes |
Table: GSEA details [plain text format]

  

Fig 2: SINGH\_SOX2\_UPREGULATED\_TRANSCRIPTS\_PMID\_28122245: Random ES distribution      
 Gene set null distribution of ES for **SINGH\_SOX2\_UPREGULATED\_TRANSCRIPTS\_PMID\_28122245**
